# Sodium 2-nitro­cinnamate dihydrate: a one-dimensional hydrogen-bonded coordination polymer

**DOI:** 10.1107/S1600536809030402

**Published:** 2009-08-08

**Authors:** Graham Smith, Urs D. Wermuth

**Affiliations:** a School of Physical and Chemical Sciences, Queensland University of Technology, GPO Box 2434, Brisbane, Qld 4001, Australia

## Abstract

The title compound *catena*-poly[aqua­sodium-μ_2_-aqua-μ_3_-2-nitro­cinnamato], [Na(C_9_H_6_NO_4_)(H_2_O)_2_]_*n*_, the sodium salt of *trans*-2-nitro­cinnamic acid, is a one-dimensional coordination polymer based on six-coordinate octa­hedral NaO_6_ centres, comprising three facially related monodentate carboxyl­ate O-atom donors from separate ligands (all bridging) [Na—O = 2.4370 (13)–2.5046 (13) Å], and three water mol­ecules (two bridging and one monodentate) [Na—O = 2.3782 (13)–2.4404 (17) Å]. The structure is also stabilized by intra-chain water–carboxyl­ate and water–nitro O—H⋯O hydrogen bonds.

## Related literature

For literature on similar compounds, see: Crowther *et al.* (2008 ▶); Kariuki *et al.* (1995 ▶); Kula *et al.* (2007 ▶); Schmidt (1964 ▶); Smith *et al.* (2006 ▶); Trividi *et al.* (2005 ▶).
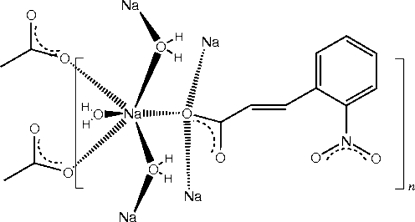


## Experimental

### 

#### Crystal data

[Na(C_9_H_6_NO_4_)(H_2_O)_2_]
*M* *_r_* = 251.17
Monoclinic, 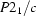

*a* = 19.4179 (7) Å
*b* = 3.6899 (2) Å
*c* = 14.8738 (7) Å
β = 92.239 (4)°
*V* = 1064.90 (9) Å^3^
*Z* = 4
Mo *K*α radiation
μ = 0.17 mm^−1^
*T* = 297 K
0.40 × 0.30 × 0.13 mm

#### Data collection

Oxford Diffraction Gemini-S CCD-detector diffractometer
Absorption correction: multi-scan (**SADABS**; Sheldrick, 1996 ▶) *T*_min_ = 0.93, *T*_max_ = 0.98
6531 measured reflections
2100 independent reflections
1626 reflections with *I* > 2σ(*I*)
*R*_int_ = 0.019

#### Refinement

*R*[*F* ^2^ > 2σ(*F* ^2^)] = 0.038
*wR*(*F* ^2^) = 0.113
*S* = 1.09
2100 reflections
170 parameters
H atoms treated by a mixture of independent and constrained refinement
Δρ_max_ = 0.30 e Å^−3^
Δρ_min_ = −0.19 e Å^−3^

### 

Data collection: *CrysAlis Pro* (Oxford Diffraction, 2009 ▶); cell refinement: *CrysAlis Pro*; data reduction: *CrysAlis Pro*; program(s) used to solve structure: *SIR92* (Altomare *et al.*, 1994 ▶); program(s) used to refine structure: *SHELXL97* (Sheldrick, 2008 ▶); molecular graphics: *PLATON* (Spek, 2009 ▶); software used to prepare material for publication: *PLATON*.

## Supplementary Material

Crystal structure: contains datablocks global, I. DOI: 10.1107/S1600536809030402/su2131sup1.cif

Structure factors: contains datablocks I. DOI: 10.1107/S1600536809030402/su2131Isup2.hkl

Additional supplementary materials:  crystallographic information; 3D view; checkCIF report

## Figures and Tables

**Table 1:** Hydrogen-bond geometry (Å, °)

*D*—H⋯*A*	*D*—H	H⋯*A*	*D*⋯*A*	*D*—H⋯*A*
O1*W*—H11*W*⋯O32^i^	0.78 (3)	2.14 (3)	2.8871 (17)	162 (2)
O1*W*—H12*W*⋯O32^ii^	0.89 (2)	1.90 (2)	2.7852 (17)	171 (2)
O2*W*—H21*W*⋯O21^iii^	0.77 (3)	2.49 (3)	3.050 (2)	131 (3)
O2*W*—H22*W*⋯O32^i^	0.91 (4)	2.04 (5)	2.882 (2)	153 (4)

Symmetry codes: (i) 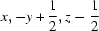; (ii) 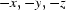; (iii) 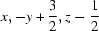.

**Fig. 1.**
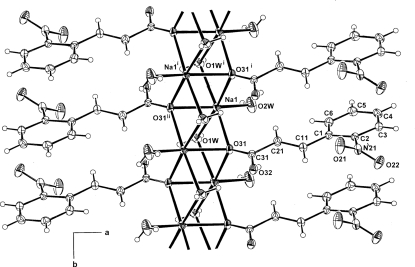
Molecular configuration and atom naming scheme for the title compound, showing the one-dimensional chain polymer structure extending along direction [010]. Displacement ellipsoids are drawn at the 50% probability level [Symmetry codes: (i) x, y + 1, z; (ii) -x, -y + 1, -z].

## supplementary crystallographic information

### Comment

Although the structures of two polymorphs of *trans*-cinnamic acid have been determined (Schmidt, 1964; Smith *et al.*, 2006), the structures of neither *trans*-2-nitrocinnamic acid [(*E*)-3-(2-nitrophenyl)propenoic acid] nor any of its alkali metal salts are known, although the dicyclohexylaminium salt has been reported (Trividi *et al.*, 2005). The only structures of alkali metal compounds of analogous ring-substituted *trans*-cinnamic acids are the sodium complexes with 2-chlorocinnamic acid (Kariuki *et al.*, 1995), 3-chlorocinnamic acid (Crowther *et al.*, 2008), and 4-hydroxy-2-methoxycinnamic acid (Kula *et al.*, 2007). We have now prepared the sodium salt of *trans*-2-nitrocinnamic acid, a dihydrate [Na(C_9_H_6_NO_4_)(H_2_O)_2_]*_n_* and its structure is reported here.

The molecular structure of the title compound is illustrated in Fig. 1. The polymeric structure is based on octahadral six-coordinate NaO_6_ centres comprising three facially related monodentate carboxylate O-donors from separate ligands (all bridging) [Na–O, 2.4370 (13)– 2.5046 (13) Å] and three water molecules (two bridging, one monodentate) [Na–O, 2.3782 (13)–2.4404 (17) Å]. These units are linked into one-dimensional coordination polymer chains which extend along direction [010] (Fig. 1). The structure is similar to that of the sodium 2-chlorocinnamate complex (Kariuki *et al.*, 1995). The polymer chains are stabilized by intra-chain water *O*–*H*···O_carboxylate_ and *O*–*H*···O_nitro_hydrogen bonds (Table 1).

In the substituted cinnamate ligand molecule, the nitro group is rotated out of the plane of the benzene ring [torsion angle C1–C2–N21–O22, 144.65 (17)°], while the carboxylate group is similarly non-coplanar [C11–C21–C31–O31, -169.51 (17)°].

### Experimental

The title compound was synthesized by heating together for 10 minutes under reflux 1 mmol quantities of *trans*-cinnamic acid [(*E*-3-(2-nitrophenyl)propenoic acid] and sodium carbonate in 50 ml of 50% ethanol-water. After concentration to *ca* 30 ml, partial rt evaporation of the hot-filtered solution gave thin colourless plate-like crystals, suitable for X-ray analysis.

### Refinement

The H-atoms of the water molecules were located in difference electron-density maps and were freely refined: O-H = 0.77 (3) - 0.91 (4) Å. The C-bound H-atoms were included in calculated positions and treated as riding atoms: C–H = 0.93 Å with *U*_iso_(H) = 1.2*U*_eq_(C).

### Crystal data

**Table:**

[Na(C_9_H_6_NO_4_)(H_2_O)_2_]	*F*(000) = 520
*M**_r_* = 251.17	*D*_x_ = 1.567 Mg m^−^^3^
Monoclinic, *P*2_1_/*c*	Mo *K*α radiation, λ = 0.71073 Å
Hall symbol: -P 2ybc	Cell parameters from 2943 reflections
*a* = 19.4179 (7) Å	θ = 3.0–28.7°
*b* = 3.6899 (2) Å	µ = 0.17 mm^−^^1^
*c* = 14.8738 (7) Å	*T* = 297 K
β = 92.239 (4)°	Plate, colourless
*V* = 1064.90 (9) Å^3^	0.40 × 0.30 × 0.13 mm
*Z* = 4	

### Data collection

**Table:**

Oxford Diffraction Gemini-S CCD-detector diffractometer	2100 independent reflections
Radiation source: Enhance (Mo) X-ray source	1626 reflections with *I* > 2σ(*I*)
graphite	*R*_int_ = 0.019
ω scans	θ_max_ = 26.0°, θ_min_ = 3.0°
Absorption correction: multi-scan (*SADABS*; Sheldrick, 1996)	*h* = −23→21
*T*_min_ = 0.93, *T*_max_ = 0.98	*k* = −4→4
6531 measured reflections	*l* = −18→17

### Refinement

**Table:**

Refinement on *F*^2^	Primary atom site location: structure-invariant direct methods
Least-squares matrix: full	Secondary atom site location: difference Fourier map
*R*[*F*^2^ > 2σ(*F*^2^)] = 0.038	Hydrogen site location: inferred from neighbouring sites
*wR*(*F*^2^) = 0.113	H atoms treated by a mixture of independent and constrained refinement
*S* = 1.09	*w* = 1/[σ^2^(*F*_o_^2^) + (0.0708*P*)^2^] where *P* = (*F*_o_^2^ + 2*F*_c_^2^)/3
2100 reflections	(Δ/σ)_max_ < 0.001
170 parameters	Δρ_max_ = 0.30 e Å^−^^3^
0 restraints	Δρ_min_ = −0.19 e Å^−^^3^

### Special details

**Table:**

Geometry. Bond distances, angles *etc*. have been calculated using the rounded fractional coordinates. All su's are estimated from the variances of the (full) variance-covariance matrix. The cell e.s.d.'s are taken into account in the estimation of distances, angles and torsion angles
Refinement. Refinement of *F*^2^ against ALL reflections. The weighted *R*-factor *wR* and goodness of fit *S* are based on *F*^2^, conventional *R*-factors *R* are based on *F*, with *F* set to zero for negative *F*^2^. The threshold expression of *F*^2^ > σ(*F*^2^) is used only for calculating *R*-factors(gt) *etc*. and is not relevant to the choice of reflections for refinement. *R*-factors based on *F*^2^ are statistically about twice as large as those based on *F*, and *R*- factors based on ALL data will be even larger.

### Fractional atomic coordinates and isotropic or equivalent isotropic displacement parameters (Å^2^)

**Table:**

	*x*	*y*	*z*	*U*_iso_*/*U*_eq_	
Na1	0.05494 (3)	0.73072 (17)	−0.06704 (4)	0.0272 (2)	
O1W	0.02635 (6)	0.2374 (3)	−0.16440 (8)	0.0319 (4)	
O2W	0.16105 (8)	0.6812 (5)	−0.14886 (12)	0.0691 (7)	
O21	0.29871 (7)	0.4625 (6)	0.31366 (9)	0.0637 (6)	
O22	0.39979 (7)	0.2280 (5)	0.32066 (10)	0.0541 (6)	
O31	0.06940 (6)	0.2430 (3)	0.04255 (8)	0.0282 (4)	
O32	0.10417 (6)	0.0743 (4)	0.18086 (8)	0.0336 (4)	
N21	0.35246 (7)	0.3769 (4)	0.27927 (10)	0.0339 (5)	
C1	0.30837 (8)	0.4562 (5)	0.12070 (11)	0.0267 (5)	
C2	0.36252 (8)	0.4742 (4)	0.18535 (11)	0.0261 (5)	
C3	0.42813 (9)	0.5880 (5)	0.16617 (12)	0.0327 (6)	
C4	0.44154 (10)	0.6987 (5)	0.08063 (14)	0.0384 (6)	
C5	0.38972 (10)	0.6860 (5)	0.01494 (13)	0.0368 (6)	
C6	0.32514 (9)	0.5634 (5)	0.03431 (12)	0.0346 (6)	
C11	0.23935 (9)	0.3141 (5)	0.13955 (12)	0.0297 (5)	
C21	0.18383 (9)	0.3638 (5)	0.08772 (13)	0.0345 (6)	
C31	0.11421 (8)	0.2155 (4)	0.10637 (11)	0.0256 (5)	
H3	0.46290	0.58950	0.21090	0.0390*	
H4	0.48520	0.78150	0.06720	0.0460*	
H5	0.39850	0.76120	−0.04320	0.0440*	
H6	0.29140	0.55150	−0.01180	0.0420*	
H11	0.23490	0.17970	0.19190	0.0360*	
H11W	0.0460 (11)	0.241 (6)	−0.209 (2)	0.055 (8)*	
H12W	−0.0168 (12)	0.157 (7)	−0.1733 (17)	0.044 (8)*	
H21	0.18810	0.50100	0.03580	0.0410*	
H21W	0.1828 (16)	0.851 (9)	−0.138 (2)	0.093 (13)*	
H22W	0.1500 (18)	0.666 (12)	−0.209 (2)	0.101 (14)*	

### Atomic displacement parameters (Å^2^)

**Table:**

	*U*^11^	*U*^22^	*U*^33^	*U*^12^	*U*^13^	*U*^23^
Na1	0.0279 (4)	0.0275 (4)	0.0260 (4)	−0.0019 (3)	−0.0005 (3)	0.0007 (3)
O1W	0.0363 (7)	0.0350 (7)	0.0244 (7)	−0.0082 (5)	0.0026 (5)	−0.0002 (5)
O2W	0.0399 (9)	0.1143 (15)	0.0532 (10)	−0.0214 (9)	0.0046 (7)	0.0098 (9)
O21	0.0357 (8)	0.1222 (15)	0.0337 (8)	0.0081 (9)	0.0067 (6)	−0.0065 (9)
O22	0.0475 (9)	0.0775 (12)	0.0364 (9)	0.0164 (7)	−0.0096 (7)	0.0112 (7)
O31	0.0220 (6)	0.0370 (7)	0.0253 (6)	−0.0013 (5)	−0.0039 (5)	0.0011 (5)
O32	0.0290 (7)	0.0461 (8)	0.0255 (7)	0.0007 (5)	0.0005 (5)	0.0056 (6)
N21	0.0266 (8)	0.0471 (9)	0.0276 (8)	−0.0020 (7)	−0.0027 (6)	−0.0022 (7)
C1	0.0235 (8)	0.0281 (9)	0.0283 (9)	0.0035 (7)	0.0004 (7)	−0.0017 (7)
C2	0.0264 (8)	0.0257 (8)	0.0260 (9)	0.0040 (7)	0.0003 (7)	−0.0020 (7)
C3	0.0255 (9)	0.0331 (10)	0.0393 (11)	−0.0010 (8)	−0.0021 (7)	−0.0033 (8)
C4	0.0299 (10)	0.0362 (10)	0.0499 (13)	−0.0071 (8)	0.0106 (9)	−0.0017 (9)
C5	0.0405 (11)	0.0374 (10)	0.0331 (11)	−0.0045 (8)	0.0097 (8)	0.0035 (8)
C6	0.0340 (10)	0.0415 (11)	0.0281 (9)	0.0024 (8)	−0.0026 (7)	0.0020 (8)
C11	0.0261 (9)	0.0353 (10)	0.0276 (9)	−0.0015 (7)	0.0001 (7)	−0.0008 (7)
C21	0.0266 (9)	0.0435 (11)	0.0334 (10)	−0.0035 (8)	−0.0004 (7)	0.0093 (8)
C31	0.0231 (8)	0.0288 (9)	0.0248 (9)	0.0039 (7)	0.0008 (7)	−0.0028 (7)

### Geometric parameters (Å, °)

**Table:**

Na1—O1W	2.3782 (13)	C1—C2	1.399 (2)
Na1—O2W	2.4404 (17)	C1—C6	1.395 (2)
Na1—O31	2.4370 (13)	C1—C11	1.476 (2)
Na1—O1W^i^	2.4162 (13)	C2—C3	1.382 (2)
Na1—O31^i^	2.5046 (13)	C3—C4	1.371 (3)
Na1—O31^ii^	2.4577 (13)	C4—C5	1.376 (3)
O21—N21	1.222 (2)	C5—C6	1.374 (3)
O22—N21	1.217 (2)	C11—C21	1.314 (3)
O31—C31	1.267 (2)	C21—C31	1.494 (2)
O32—C31	1.247 (2)	C3—H3	0.9300
O1W—H11W	0.78 (3)	C4—H4	0.9300
O1W—H12W	0.89 (2)	C5—H5	0.9300
O2W—H21W	0.77 (3)	C6—H6	0.9300
O2W—H22W	0.91 (4)	C11—H11	0.9300
N21—C2	1.463 (2)	C21—H21	0.9300
			
O1W—Na1—O2W	79.67 (5)	C2—C1—C6	115.06 (15)
O1W—Na1—O31	81.96 (4)	C2—C1—C11	123.37 (15)
O1W—Na1—O1W^i^	100.64 (4)	C6—C1—C11	121.46 (15)
O1W—Na1—O31^i^	172.53 (5)	N21—C2—C1	121.34 (14)
O1W—Na1—O31^ii^	85.02 (4)	N21—C2—C3	115.49 (14)
O2W—Na1—O31	101.58 (6)	C1—C2—C3	123.16 (15)
O1W^i^—Na1—O2W	86.43 (5)	C2—C3—C4	119.49 (17)
O2W—Na1—O31^i^	107.80 (5)	C3—C4—C5	119.26 (18)
O2W—Na1—O31^ii^	158.48 (6)	C4—C5—C6	120.69 (18)
O1W^i^—Na1—O31	171.93 (5)	C1—C6—C5	122.29 (16)
O31—Na1—O31^i^	96.60 (4)	C1—C11—C21	124.71 (17)
O31—Na1—O31^ii^	91.04 (4)	C11—C21—C31	124.59 (17)
O1W^i^—Na1—O31^i^	79.83 (4)	O31—C31—C21	115.57 (14)
O1W^i^—Na1—O31^ii^	81.62 (4)	O32—C31—C21	119.48 (15)
O31^i^—Na1—O31^ii^	87.68 (4)	O31—C31—O32	124.96 (15)
Na1—O1W—Na1^iii^	100.64 (5)	C2—C3—H3	120.00
Na1—O31—C31	128.20 (10)	C4—C3—H3	120.00
Na1—O31—Na1^iii^	96.60 (5)	C3—C4—H4	120.00
Na1—O31—Na1^ii^	88.96 (4)	C5—C4—H4	120.00
Na1^iii^—O31—C31	118.92 (10)	C4—C5—H5	120.00
Na1^ii^—O31—C31	122.84 (10)	C6—C5—H5	120.00
Na1^iii^—O31—Na1^ii^	92.32 (4)	C1—C6—H6	119.00
H11W—O1W—H12W	112 (2)	C5—C6—H6	119.00
H21W—O2W—H22W	111 (4)	C1—C11—H11	118.00
O21—N21—C2	118.98 (14)	C21—C11—H11	118.00
O22—N21—C2	117.87 (14)	C11—C21—H21	118.00
O21—N21—O22	123.06 (16)	C31—C21—H21	118.00
			
O2W—Na1—O1W—Na1^iii^	95.66 (6)	O31—Na1—O31^ii^—C31^ii^	−136.41 (11)
O31—Na1—O1W—Na1^iii^	−7.75 (5)	Na1—O31—C31—O32	145.52 (13)
O1W^i^—Na1—O1W—Na1^iii^	180.00 (6)	Na1—O31—C31—C21	−35.11 (19)
O31^ii^—Na1—O1W—Na1^iii^	−99.51 (5)	Na1^iii^—O31—C31—O32	−87.48 (18)
O1W—Na1—O31—C31	142.67 (13)	Na1^iii^—O31—C31—C21	91.89 (14)
O1W—Na1—O31—Na1^iii^	7.39 (4)	Na1^ii^—O31—C31—O32	26.8 (2)
O1W—Na1—O31—Na1^ii^	−84.82 (4)	Na1^ii^—O31—C31—C21	−153.81 (11)
O2W—Na1—O31—C31	65.02 (14)	O21—N21—C2—C1	−38.6 (2)
O2W—Na1—O31—Na1^iii^	−70.26 (6)	O21—N21—C2—C3	140.47 (18)
O2W—Na1—O31—Na1^ii^	−162.47 (5)	O22—N21—C2—C1	144.65 (17)
O31^i^—Na1—O31—C31	−44.72 (13)	O22—N21—C2—C3	−36.2 (2)
O31^i^—Na1—O31—Na1^iii^	180.00 (4)	C6—C1—C2—N21	178.85 (15)
O31^i^—Na1—O31—Na1^ii^	87.79 (4)	C6—C1—C2—C3	−0.2 (3)
O31^ii^—Na1—O31—C31	−132.51 (13)	C11—C1—C2—N21	−4.8 (3)
O31^ii^—Na1—O31—Na1^iii^	92.21 (5)	C11—C1—C2—C3	176.18 (17)
O31^ii^—Na1—O31—Na1^ii^	0.00 (3)	C2—C1—C6—C5	−1.5 (3)
O1W—Na1—O1W^i^—Na1^i^	−180.00 (6)	C11—C1—C6—C5	−177.99 (17)
O2W—Na1—O1W^i^—Na1^i^	−101.22 (6)	C2—C1—C11—C21	164.51 (18)
O2W—Na1—O31^i^—Na1^i^	75.56 (6)	C6—C1—C11—C21	−19.4 (3)
O2W—Na1—O31^i^—C31^i^	−65.26 (12)	N21—C2—C3—C4	−177.36 (16)
O31—Na1—O31^i^—Na1^i^	180.00 (3)	C1—C2—C3—C4	1.7 (3)
O31—Na1—O31^i^—C31^i^	39.19 (12)	C2—C3—C4—C5	−1.6 (3)
O1W—Na1—O31^ii^—Na1^ii^	81.83 (4)	C3—C4—C5—C6	−0.1 (3)
O1W—Na1—O31^ii^—C31^ii^	−54.58 (11)	C4—C5—C6—C1	1.7 (3)
O2W—Na1—O31^ii^—Na1^ii^	126.42 (15)	C1—C11—C21—C31	179.16 (16)
O2W—Na1—O31^ii^—C31^ii^	−10.0 (2)	C11—C21—C31—O31	−169.51 (17)
O31—Na1—O31^ii^—Na1^ii^	0.00 (5)	C11—C21—C31—O32	9.9 (3)

Symmetry codes: (i) *x*, *y*+1, *z*; (ii) −*x*, −*y*+1, −*z*; (iii) *x*, *y*−1, *z*.

### Hydrogen-bond geometry (Å, °)

**Table:**

*D*—H···*A*	*D*—H	H···*A*	*D*···*A*	*D*—H···*A*
O1W—H11W···O32^iv^	0.78 (3)	2.14 (3)	2.8871 (17)	162 (2)
O1W—H12W···O32^v^	0.89 (2)	1.90 (2)	2.7852 (17)	171 (2)
O2W—H21W···O21^vi^	0.77 (3)	2.49 (3)	3.050 (2)	131 (3)
O2W—H22W···O32^iv^	0.91 (4)	2.04 (5)	2.882 (2)	153 (4)
C11—H11···O21	0.93	2.39	2.846 (2)	110

Symmetry codes: (iv) *x*, −*y*+1/2, *z*−1/2; (v) −*x*, −*y*, −*z*; (vi) *x*, −*y*+3/2, *z*−1/2.
